# A study on causes of cattle liver condemnation at an abattoir in Omdurman area, Khartoum State, Sudan

**DOI:** 10.1186/s12917-021-02766-4

**Published:** 2021-01-28

**Authors:** Darien Kheder, Ali Mohamed

**Affiliations:** Department of Veterinary Preventive Medicine Public Health, Faculty of Veterinary Medicine, University of Khartoum, Khartoum North, Sudan

**Keywords:** Meat inspection, Post-mortem, Zoonotic disease, Liver condemnation, Fasciolosis

## Abstract

**Background:**

Information obtained from abattoirs on the causes of liver condemnation is important in preventing the spread of diseases and for promoting food security. The current study reviews three years (2009 to 2011) postmortem inspection records of cattle slaughtered at an abattoir in Omdurman, Khartoum State, Sudan. The aim was to determine the prevalence of diseases and conditions that lead to liver condemnation.

**Results:**

From a total of 234,175 cattle slaughtered, 8,910 (3.8%) livers were condemned due to several diseases/conditions mainly fasciolosis, cysticercosis, necrosis, abscess, calcification, hemorrhages, liver cirrhosis, hydatidosis, and other miscellaneous causes. Collectively, fasciolosis was the leading cause of liver condemnation and was responsible for 51.6 % of total liver condemnations followed by necrosis (18.6%), and cysticercosis (13.5%).

**Conclusions:**

Because of their zoonotic nature, the observed high frequency of some detected diseases/conditions is thought to pose a public health risk among consumers. This survey could be used as a regional baseline for future monitoring of control programmers against these liver diseases.

## Background

In Sudan the livestock sector plays a critical role in the economy as well as the welfare of the whole population with a population estimated at 39.5 million head and exhibiting an average annual growth rate of 3.6 %. Most cattle population in Sudan owned by pastoralists and distributed in two major regions; Western Sudan, the homeland for Baggara cattle; Mid-Sudan, the homeland for the Kenana and Butana breeds [1].

Livestock production in Sudan plays a vital role in national food security and hard currency income generation from export thereby improving socio-economic status. Internationally, food safety has become a subject of policy importance [2]. Meat inspection at the abattoir is a crucial need for food safety and disease control. It is one of the most widely implemented and longest-running systems of surveillance that involves the screening of animals and meat for wholesomeness for human consumption [3]. Meat inspection plays several roles in safeguarding the public health e.g. removing gross abnormalities from meat and its products, preventing the distribution of contaminated meat and assisting in the detection and eradication of certain livestock diseases and potentially zoonotic infections. Endemic and exotic diseases that result in mortality and morbidity are the main constraint to efficient livestock production that decreases the available food from farming systems. The importance of food safety for human health has been widely known. The safety of foods of animal origin is particularly relevant because the large majority of food borne diseases come from poultry, eggs, meat, milk and dairy products and fish [4]. As Sudan’s population increases, the demand for limited food resources will increase to ensure food security, consequently, the necessity for documentation of causes of food losses thanks to meat condemnation will arise. Moreover, the implications of zoonotic diseases affirm the important role that information obtained from meat inspection plays in the enhancement of public health and food safety [4, 5]. Prevalence of infection with meat-borne parasites in humans and livestock in Sudan is not accessible. Strategies for routine diagnosis, checking or recording of those infections are insufficient, or not existing. Consequently, researchers should be encouraged to participate and establish innovative ways and means to control these diseases [6]. Moreover, bacterial diseases such as bovine tuberculosis (4.5 %) are present in slaughtered cattle with caseous lesions [7]. Micrococcus, Bacillus, Staphylococcus, Corynebacterium, Propionibacterium, Actinomyces, Pseudomonas, Escherichia coli, Corynebacterium, Aerococcus, Klebsella, and Moraxella were isolated from contaminated meat in several abattoirs in Khartoum State, Sudan [8–10].

Identification and quantification of causes of liver condemnation aimed at preventing further liver losses during meat inspection. Liver condemnations represent a reduction in available food resources. At routine post-mortem meat inspection, the liver is condemned due to preventable lesions/diseases detected. Many studies in Africa have shown a high prevalence of preventable parasitic and bacterial zoonosis [11–13], demonstrating the lack of a proper herd health program necessary for the promotion of animal health in farms. Moreover, data on beef condemnation could provide information on the epidemiology of livestock diseases and indicate the extent of public exposure to certain zoonotic diseases. Previous studies revealed that the greatest losses of meat were due to the condemnation of livers, followed by lungs and carcasses, in descending order [14, 15]. They attributed the losses to fasciolosis, hydatididosis, and cysticercosis. The current study, aimed at identifying the major causes of cattle liver condemnation in cattle livers intended for human consumption in Khartoum state, Sudan.

## Results

### Reasons for liver condemnation

Data about the number of slaughtered cattle and condemned livers over three years (2009–2011) are displayed as numbers and percentages in Table 1. The Retrospective Study revealed the leading cause of liver condemnation of the total number of condemned livers as fasciolosis (45.5 %), necrosis (25.6 %), cysticercosis (15.8 %), and abscesses (7.6 %) in 2009. In 2010, fasciolosis (45.3 %), necrosis (22.0 %), cysticercosis (14.6 %), and abscesses (8.0 %) were the main reasons for liver rejection at the abattoir. In 2011, livers were mostly condemned due to fasciolosis (64.4 %), cysticercosis (10.3 %), necrosis (8.9 %), and abscesses (6.3 %). Calcification (3.1 %), fatty change (2.4 %), and fibrosis (1.7 %) were other causes of liver rejection during the study period as represented in Table 1.

**Table 1:** Annual prevalence of causes of liver condemnation (2009–2011)

		Number (%) of Condemned Organs during the Three Years							
		2009		2010		2011		Total	
		Number	%	Number	%	Number	%	Number	%
**Causes of liver condemnation**	**Fasciola**	937	45.5	1615	45.3	1751	64.4	4303	51.6
	**C. bovis**	326	15.8	519	14.6	280	10.3	1125	13.5
	**Necrosis**	527	25.6	783	22.0	242	8.9	1552	18.6
	**Abscess**	157	7.6	285	8.0	172	6.3	614	7.4
	**Fibrosis**	8	0.4	83	2.3	54	2.0	145	1.7
	**Calcification**	27	1.3	112	3.1	119	4.4	258	3.1
	**Hemorrhage**	6	0.3	28	0.8	23	0.8	57	0.7
	**Cirrhosis**	1	0.0	1	0.0	6	0.2	8	0.1
	**Congestion**	11	0.5	5	0.1	7	0.3	23	0.3
	**Fatty change**	43	2.1	113	3.2	48	1.8	204	2.4
	**Adhesion**	7	0.3	4	0.1	5	0.2	16	0.2
	**Hydatid cyst**	6	0.3	13	0.4	6	0.2	25	0.3
	**Jaundice**	0	0.0	0	0.0	2	0.1	2	0.0
	**Tuberculosis**	3	0.1	2	0.1	5	0.2	10	0.1
**Total condemned**		**2059**	**3.0**	**3566**	**4.5**	**2720**	**3.2**	**8345**	**3.6**
**Total slaughtered**		**68232**		**80072**		**85871**		**234175**	

### Seasonal trend and occurrence of the major pathologic lesions

**Fig. 1:**
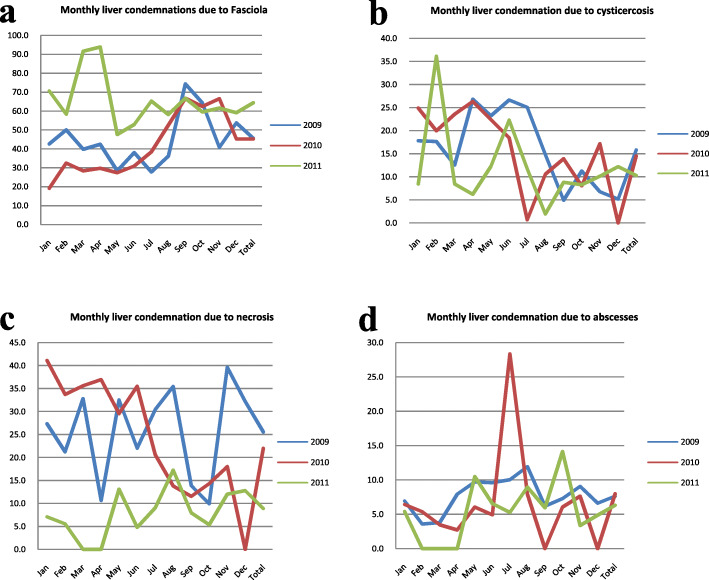
Trend of major liver condemnation 2009–2011 due to; **a**: Fasciola, **b**: Cysticercosis, **c**: Necrosis, **d**: Abscesses

A three-year summary to compare the trend in the seasonal prevalence of the major pathologic lesions has also noted that the prevalence of the fasciolosis and cysticercosis displayed some seasonal patterns, while such trends were not observed for neither necrosis nor abscesses as shown in Fig. 1(a, b, c and d). For example, the prevalence of fasciolosis was highest between Augest and October except for the year 2011 where no or a few data about other causes of liver condemnation but fasciolosis were recorded. On the other hand, cysticercosis was observed to be more prevalent between March to July.

## Discussion

This study illustrated the usefulness of meat inspection records in monitoring disease conditions and demonstrated possible seasonal trends of the investigated causes of condemnation. Although, abattoir surveys have limitations, they are an economical way of gathering information on livestock diseases especially in a developing country like Sudan. Postmortem meat inspection is intended to spot and remove from the food chain all carcasses/offal those present grossly identifiable abnormalities which may affect the safety security, and wholesomeness of the meat product [16, 17].

The ongoing climate change especially warm winters and increased humidity is providing favorable environment parasites and increase chances of infection in livestock leading to poor health and decreased production. Thus, there is an urgent need of improved surveillance, diagnosis and systematic study of parasitic diseases brought about by climate change for efficient disease preventive strategies.

The present study investigated the main causes of bovine liver condemnations using data from at an abattoir in Omdurman for a period of three years from January 2009 to December 2011. Al-Mowailih is the central market and one amongst the main marketing channels for animals in Sudan. Therefore, the collected data is representative of the major production areas of the country. The numbers of livers condemned due to specific causes were obtained and analyzed to identify the major causes of liver condemnations in Sudan.

The results of the current study came in a harmony with other studies from different areas of Sudan indicated that the higher economic loss were a condemnation of edible organs and carcasses due to parasitic disease [14, 15].

Liver condemnations have been studied by several researchers in different African countries namely Ethiopia, Tanzania, Zambia and Zimbabwe and shown a high prevalence of preventable zoonosis, demonstrating a weakness in the proper herd health programs necessary for the promotion of animal health in the various farms where these animals are bred [11, 12, 18–20]. The situation in Sudan may be more critical if not similar and the animal health programs need to be improved to cope with the international standard, the increasing animal population and increasing national and global food consumption rates.

Losses from liver condemnation were generally reported to associate with infection of public health importance [21]. Livers were the most rejected organ at post-mortem meat inspection as has also been reported in other studies in some African countries [22–24]. The findings of those studies were to some extent came in agreement with the present study which may be thanks to ecological, climate, and management practices similarities between the study areas. In agreement with [12, 22], fasciolosis and cysticercosis were the major causes of liver condemnation. Fasciolosis is an important disease in an economical point of view as it causes huge financial losses to butchers, farmers and consumers in forms of liver condemnation, poor quality carcass, reduction in growth rate, decrease conception rate, less productivity, and mortality [25, 26]. The high prevalence of liver flukes may well be explained by the endemicity of the parasite in Sudan due to the high rainfall that favors the proliferation of the snail intermediate hosts. Moreover, seasonal trend of fasciolosis was revealed from the studied data, since the prevalence was higher during the wet season (August- November).

Cysticercosis is economically important due to the associated meat condemnations. Although occurring at a far lower prevalence, it is a zoonotic parasite of public health concern. Prevention and control measures especially the utilization of toilets for defecation, treatment of infected individuals followed by regular washing of hands after toilet use, must be implemented to stop further losses [27]. Seasonal trend was also noticed in the prevalence of cysticercosis. The prevalence was higher during summer season (March - July) which is considered to be hot and dry.

Method of meat inspection, inspector’s experience [12, 28, 29], farm management differences, sampling method, lesion location and degree of degenerated cysts [30, 31], additionally to other factors might significantly contribute to variations within the recorded prevalence of bovine cysticercosis. The prevalence of cysticercosis obtained in the current study could be an underestimation of the true prevalence due to improper recording (partial condemnation is not recorded) and an undefined number of livers condemned for abscessation. This emphasizes the requirement for investigating the causes of liver abscesses so as to implement prevention and control measures.

Data for records also included other causes of liver condemnations like fatty change, fibrosis, calcification, hemorrhage, cirrhosis, congestion, adhesion, hydatid cyst, jaundice, and tuberculosis. These causes may be associated with infectious causes that require further investigation to identify the etiology and to reduce the incidence and for further implementation of suitable preventive and control measures [28, 32]. The rarity of some of these lesions would suggest that they are likely to be of minor concern and would be expected to appear only sporadically with no evidence of seasonality.

A high prevalence of hydatidosis cattle livers (3 % of 2,368) was reported in Sudan [33], while a lower percentage was obtained in the current study (0.3 % of 234,175) the difference in the prevalence rate may be attributed to an improvement in the veterinary services, the location of the study, livestock husbandry, abundance of infected definitive host, nature of the pasture and grazing patterns of the slaughtered cattle.

The present study revealed that fasciolosis, cysticercosis, necrosis, and abscesses were the main causes of liver condemnation in cattle leading to considerable economical loss of meat. Eradication of those diseases requires cooperation between the general public health and official veterinary authorities. It is recommended for each country to establish a public health education for farmers and to implement farmer based organization extension. Farmer’s education is necessary to avoid eating raw meat, proper disposal of condemned organs, cattle management system, treatment of animals with anti-helminthic drugs and grazing management of animals during the dry season to avoid the access of the animals to the parasite’s eggs.

Generally, retrospective studies possess some limitations that could result in an underestimated prevalence of liver diseases due to several reasons e.g. meat inspector’s judgment errors, only clinically healthy animals are passed for slaughter, relying on gross pathological lesions for the diagnosis of diseases lead to condemnation of livers and general substandard record keeping. Furthermore, localized or partial infection of livers that might have been passed as fit for human consumption after trimming of the affected parts and not included in the slaughterhouse records.

## Conclusions

The current study emphasizes that determining the causes of meat condemnation is important for developing an effective control strategies. Moreover, retrospective study is identified as a basic tool for continuous monitoring and evaluation of meat inspection to reduce economic losses and insuring food security. Strategic measures need to be implemented to control parasitic infections in cattle, dogs, and humans in order to reduce the incidence of parasitic condemnations as well as the public health implications of zoonoses. The results of meat inspection is recommended to be regularly communicated to the responsible veterinarians and public health officials for tracing back diseases in the affected areas and to control of the specified diseases in animals and humans.

Although costly, proficiency tests are needed for adequate meat inspection and hygiene by supporting diagnostic laboratories that would greatly increase the amount of important information obtainable from abattoirs and contribute to combating controllable and notifiable diseases as well as zoonotic diseases.

## Methods

### Study area and animals

Omdurman is the most populated city in Sudan and Khartoum State, lying on the western banks of the River Nile. It features a hot arid climate, with an average of a little over 155 millimeters of precipitation per year. Based on annual mean temperatures, the city is one of the hottest major cities in the world.

The current study was conducted using data from an abattoir located in west Omdurman in the vicinity of the country’s main livestock market of Al-Mowailih cattle market (established in 1981), a crossing center connected with production regions all over the country. The abattoir is considered one of Sudan’s large-scale integrated investments for local and export production of red meat. The study animals were cattle brought to the abattoir for slaughter from different production regions in Sudan. The daily cattle slaughter in the slaughterhouse is estimated to be 200–300 head/day, although the capacity of the slaughterhouse was three times more than that.

### Study design and data collection

This study involved the retrieval of slaughter records from three years from 2009 to 2011. Data were obtained by the help of an experienced team of veterinarians. The collected information included the number of cattle slaughtered, the number of condemned livers, and causes for each condemnation. As a means of quality control of data, all records with no proper diagnosis of liver lesions and ambiguous information were excluded from the study. Routine meat inspection was carried out by veterinarians.

### Inspector’s judgment

Condemnation depends on the inspector’s experience. Thus, the animals were evaluated by veterinary requirements for the production of safe meat. The classification, “condemned” means that, even after special treatment, the beef (liver) does not fulfill veterinary requirements for the production of safe meat and organs (e.g. because of lesions making the meat unsuitable for human consumption, sensorial changes and of unknown origin). In cases of meat classified as condemned, veterinary inspectors recorded numbers of lesions due to several diseases, conditions, and changes in sensorial parameters. No information about partial condemnation was recorded by the responsible veterinarians.

### Data collection and analysis

The study was conducted using the available data of meat inspection from the abattoir for the period from January 2009 to December 2011. Records of monthly and annually liver returns from the abattoirs were recorded concerning the number of cattle slaughtered and the corresponding number of livers condemned. The prevalence of diseases/conditions was monthly and annually calculated as percentages of the liver condemned during the same period. The overall prevalence for the three years (2009–2011) was also determined. Data obtained were entered, validated and calculated in Microsoft Excel 2007 spreadsheet and the proportions (%) of lesions were calculated considering the number of rejected livers due to a specific cause, against the total number of condemned livers.

## Data Availability

The datasets used and/or analyzed during the current study are available from the corresponding author on reasonable request.
