# Correction to: A study on causes of cattle liver condemnation at an abattoir in Omdurman area, Khartoum State, Sudan

**DOI:** 10.1186/s12917-021-03004-7

**Published:** 2021-09-06

**Authors:** Darien Kheder Ali Mohamed

**Affiliations:** Department of Veterinary Preventive Medicine Public Health, Faculty of Veterinary Medicine, University of Khartoum, Khartoum North, Sudan

**Correction to: BMC Vet Res 17, 58 (2021)**

**https://doi.org/10.1186/s12917-021-02766-4**

The original article [1] contained an error in presentation of author, Darien Kheder Ali Mohamed’s name which has since been corrected.
